# Probing the canonicity of the Wnt/Wingless signaling pathway

**DOI:** 10.1371/journal.pgen.1006700

**Published:** 2017-04-03

**Authors:** Alexandra Franz, Daria Shlyueva, Erich Brunner, Alexander Stark, Konrad Basler

**Affiliations:** 1 Institute of Molecular Life Sciences, University of Zurich, Zurich, Switzerland; 2 Research Institute of Molecular Pathology (IMP), Vienna Biocenter (VBC), Campus-Vienna-Biocenter 1, Vienna, Austria; Stanford University School of Medicine, Howard Hughes Medical Institute, UNITED STATES

## Abstract

The hallmark of canonical Wnt signaling is the transcriptional induction of Wnt target genes by the beta-catenin/TCF complex. Several studies have proposed alternative interaction partners for beta-catenin or TCF, but the relevance of potential bifurcations in the distal Wnt pathway remains unclear. Here we study on a genome-wide scale the requirement for Armadillo (Arm, *Drosophila* beta-catenin) and Pangolin (Pan, *Drosophila* TCF) in the Wnt/Wingless(Wg)-induced transcriptional response of *Drosophila* Kc cells. Using somatic genetics, we demonstrate that both Arm and Pan are absolutely required for mediating activation and repression of target genes. Furthermore, by means of STARR-sequencing we identified Wnt/Wg-responsive enhancer elements and found that all responsive enhancers depend on Pan. Together, our results confirm the dogma of canonical Wnt/Wg signaling and argue against the existence of distal pathway branches in this system.

## Author summary

Our manuscript addresses the question of whether either of the canonical transduction components, beta-catenin or TCF, can be bypassed when the Wnt pathway is activated. By using somatic cell genetics in *Drosophila* cells (via CRISPR/Cas9 editing) in combination with RNA-seq and STARR-seq (Self-transcribing-active-regulatory-region-sequencing) as functional read-outs, we provide firm evidence against the existence of distal branches in the Wnt pathway.

## Introduction

Wnt proteins are highly conserved signaling molecules specifying the fate and behavior of cells in multicellular animals ranging from nematodes to humans [1]. They play crucial roles in embryogenesis, pattern formation and tissue homeostasis during development and in adult life. Therefore it is not surprising that aberrant Wnt signaling has been found to be implicated in many human diseases [2].

Following the identification of Wnt proteins nearly 40 years ago [3–5] genetic and biochemical studies have revealed mechanistic details of how the signaling cascade operates when cells receive a Wnt signal [for review see 6]. As a consequence of Wnt/Wg proteins binding their cognate receptors, beta-catenin is no longer marked for degradation and accumulates in the cytoplasm and nucleus [7–10]. In the prevailing model, TCF is targeted through its DNA binding domain to Wnt-responsive elements (WREs) in the promoters or enhancers of target genes [11] and initiates the transcription of Wnt/Wg-responsive genes when complexed with beta-catenin. In the absence of Wnt/Wg ligand, beta-catenin is phosphorylated and degraded while TCF is bound by transcriptional repressors, such as Groucho and Coop [12–15]. In contrast to the well-studied mechanism of gene activation, the mechanisms by which beta-catenin and TCF promote target gene repression are not well understood [16]. Several reports suggest that, in addition to beta-catenin and TCFs, other factors are involved in Wnt-mediated repression, such as Prop1, Mad or Zic [17–19]. Furthermore it is not clear, in which context alternative [20] or traditional TCF binding sites are used for transcriptional repression [21–23].

A recent study showed that TCF4 is a predominant factor in mediating the Wnt response and for recruiting beta-catenin to DNA [24], however ongoing research on the Wnt signaling pathway has repeatedly demonstrated that beta-catenin as well as TCF interacts with various other proteins. Yet it remains to be determined, whether alternative transcriptional complexes also regulate the expression of Wnt/Wg target genes. For example, an interaction between beta-catenin and FOXO-transcription factors in mouse and DLD-1 human colon carcinoma cells has been demonstrated resulting in the activation of genes involved in oxidative stress and colon cancer metastasis [25–27]. Furthermore in mouse embryonic stem cells it was shown that beta-catenin forms a complex with Oct4 to promote Oct4-driven transcription and pluripotency [28]. In addition, studies in *Xenopus* reported an interaction between beta-catenin and Sox17, promoting expression of Sox17 target genes [29], and more recently it was suggested that beta-catenin complexes with YAP1 and TBX5 in human cancer cell lines [30]. In addition, alternative binding partners have also been reported for TCF, such as Plakoglobin or Mad [31, 18].

In this study, we address the question of whether alternative routes exist that bypass beta-catenin or TCF to promote the transcription of Wnt/Wg target genes in *Drosophila* cells. Using cells that lack either Arm or Pan and functional read-outs (i.e. RNA-seq and STARR-seq), we show that both, Arm and Pan, are absolutely required for target gene activation and repression. Consistent with these findings, we further demonstrate that Wnt/Wg-responsive enhancers also require Pan, arguing against the existence of distal branches in the Wnt signaling pathway.

## Results

### Genome-wide identification of Wnt/Wg target genes by RNA-sequencing

**Fig 1:**
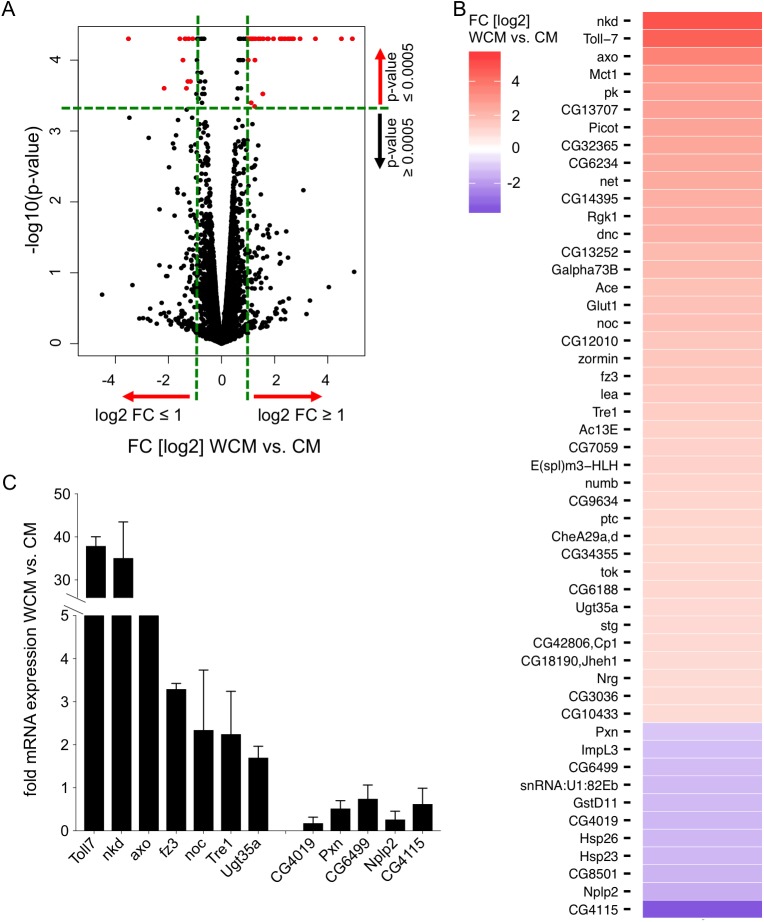
Identification of Wnt/Wg-responsive genes. (A) Volcano plot showing the log2 fold change (x-axis) and statistical significance (y-axis; -log10 p-value) of Wnt/Wg-responsive genes. 51 genes were significantly differently expressed after WCM-treatment (red dots), 40 genes showed an up-regulation (positive targets), 11 genes were down-regulated (negative targets) after WCM treatment. Black dots represent all genes that showed an altered expression profile. (B) Heatmap showing the log2 fold change (FC) of expression of the 51 Wnt/Wg-responsive genes in wild-type cells (WT). The expression profile of positive target genes is depicted in red, the expression profile of negative targets is in blue. (C) Confirming of candidate genes using qRT-PCR. *Drosophila* Kc cells were stimulated with WCM or CM for 24 h. Analysis of expression profiles of candidate target genes (7 positive and 5 negative) after treatment versus control confirmed their induction after WCM stimulation.

Next-generation RNA-sequencing (RNA-seq) was used to identify and quantify the expression of target genes of the Wnt/Wg signaling pathway in *Drosophila* Kc167 cells. Cells were treated either with Wg-enriched medium (referred to as Wingless-conditioned medium, WCM; [32]), or control-conditioned medium (CM) lacking the Wg ligand. Wg-responsive genes were determined by statistical analysis of gene expression levels in treated samples versus control samples, according to a protocol described in [33]. In order to determine a high confidence set of Wnt/Wg targets, genes had to pass the following selection criteria: exhibit a significantly altered expression profile (WCM vs CM, p-value ≤ 0.0005) and an at least two-fold change of expression upon Wg stimulation (Fig 1A). WCM-treatment resulted in the robust induction of 51 genes. Among them we found previously identified Wnt/Wg target genes such as *naked cuticle* (*nkd*), *CG6234*, *frizzled 3* (*fz3*) and *Peroxidasin* (*Pxn*) [34–36, 20], confirming our quality filters. 40 genes were at least two fold up-regulated (positive targets) and 11 genes two fold down-regulated (negative targets) (Fig 1B). 7 positive and 5 negative candidate target genes were confirmed by qRT-PCR (Fig 1C). This high confidence set of Wnt/Wg target genes was used to systematically elucidate potential beta-catenin or TCF-independent branches of Wnt/Wg signaling.

### An absolute requirement for Armadillo for activation and repression of Wg target genes

**Fig 2:**
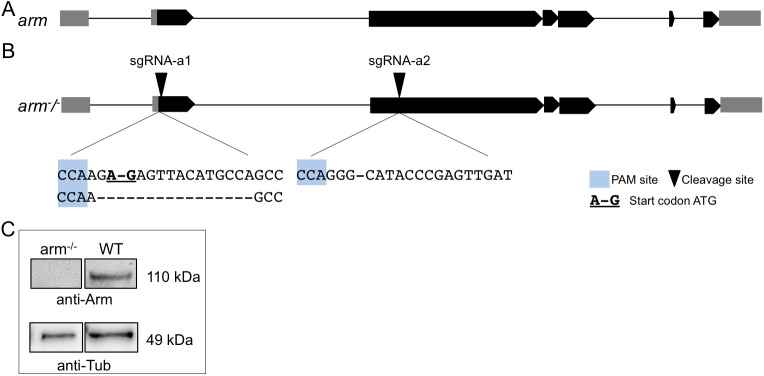
Mutagenesis of the *arm* gene. (A) Schematic of the *arm* gene-locus. Untranslated regions (UTR) are indicated in grey boxes, translated exons in black. (B) CRISPR targeting strategy: Target sites of both sgRNAs are represented by black triangles. The PAM site is highlighted in blue. Sequences as they are present in arm^-/--AFII7/8^ (arm^-/-^) cells are depicted below. Bold and underlined nucleotides represent the TSS. (C) Western blot analysis using an α-Arm antibody on lysates from wild-type (WT) and arm^-/--AFII7/8^ (arm^-/-^) cells. As expected the arm^-/--AFII7/8^ (arm^-/-^) cells are devoid of Arm protein. Tubulin was used as loading control.

To investigate whether Arm can be bypassed via alternative branches of the pathway, we generated *arm* knockout cells using the CRISPR/Cas9 technology as described by Bassett and colleagues [37]. In order to generate *Drosophila arm* null mutant cells we used sgRNAs targeting two different exons that are present in all transcript variants (Fig 2A and 2B). sgRNA-a1 on the reverse strand targets the translational start site residing in exon 2. sgRNA-a2 targets a site in the third exon. The presence of CRISPR-induced mutations generated by NHEJ (non-homologous end joining) was assessed by sequencing of the PCR products spanning the sgRNA target sites (see Material and Methods). The analysis revealed that most of the alleles had indel mutations at the expected cleavage sites, some of which lead to the deletion of the translational start site or to frameshifts in exon 3. To generate an *arm*^*-/-*^ cell line, we carried out serial dilutions and searched for cell populations that carried previously identified mutations using allele-specific primers as described in [38]. In this way, we isolated an *arm* null mutant cell line (named arm^-/--AFII7/8^) which was a homogenous cell population (see Material and Methods) carrying a deletion of either one or sixteen nucleotides in the second exon, each of them destroys the START codon (ATG), and a deletion of one nucleotide in the third exon (Fig 2B). Importantly no wild-type alleles were present. These mutations, affecting both *arm* alleles, result in frameshift mutations introducing a premature termination codon that should trigger nonsense-mediated mRNA decay (NMD) [39] (S1A Fig). We confirmed the complete loss of Arm protein in arm^-/--AFII7/8^ cells by Western blot analysis (Fig 2C, S1B Fig).

**Fig 3:**
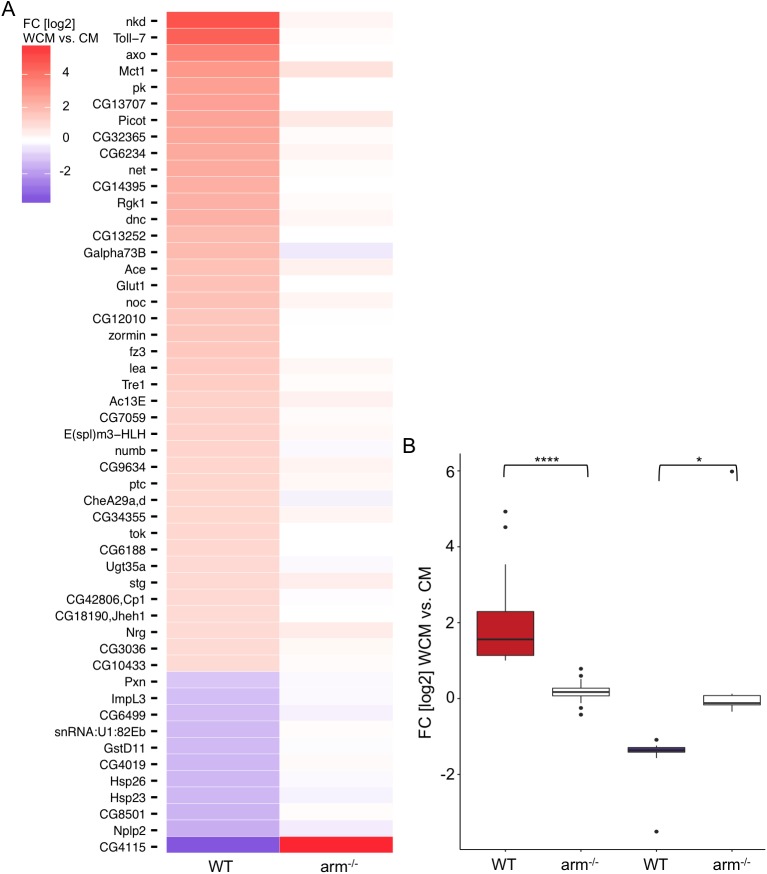
Gene expression analysis of Wg/Wnt target genes in arm^-/--AFII7/8^ cells. (A) Heat map of Wnt/Wg target genes showing their log2 fold change (FC) of expression after to before WCM treatment in wild-type (WT) and arm^-/--AFII7/8^ (arm^-/-^) cells. The genes are listed according to the strength of the induction of their expression in WT cells. Strongest up-regulated genes are on top. Up-regulated genes are shown in red, down-regulated genes in blue, no expression in white. (B) Boxplots showing the difference in gene activity for up- and down-regulated genes after WCM stimulation in wild-type (WT) and arm^-/--AFII7/8^ (arm^-/-^) cells. Paired t-test: * ≤ 0.05, *** ≤ 0.0001.

Next we investigated whether Arm is absolutely required for the Wnt/Wg-driven transcriptional output. To that end arm^-/--AFII7/8^ cells were treated either with WCM or CM and target gene responses were monitored by RNA-seq. We found that the induction of the positive Wnt/Wg target genes is dependent on Arm, since their expression was not changed in *arm* null mutant cells. Similarly all negative target genes are no longer repressed in arm^-/--AFII7/8^ cells (Fig 3A and 3B). These results demonstrate that Arm is absolutely necessary for both, activation and repression of identified Wnt/Wg targets. We confirmed our results with qRT-PCR analysis of 11 candidate targets genes (S2 Fig).

### Requirement of Pangolin for transcriptional regulation of Wg target genes

**Fig 4:**
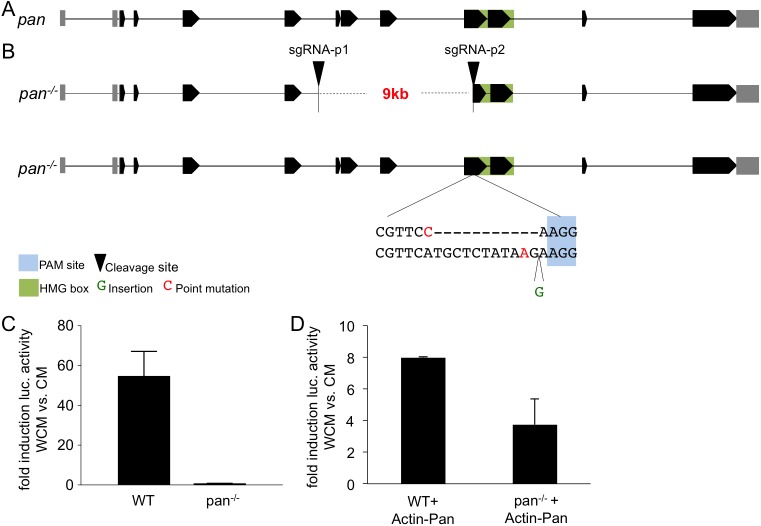
Mutagenesis of the *pan* gene. (A) Schematic of the *pan* gene-locus. Untranslated regions (UTR) are indicated in grey boxes, translated exons in black. HMG box in green. (B) CRISPR targeting strategy: Target sites of both sgRNAs are represented by black triangles. The PAM site is highlighted in blue, the HMG box in green. Sequences as they are present in the pan^-/--AF1AD26^ (pan^-/-^) cells are depicted below. (C) Wild-type (WT) and pan^-/--AF1AD26^ (pan^-/-^) cells were transfected with the *wingful* luciferase reporter expression vector and Renilla expression vector 24 h prior stimulation with WCM (as control CM was used). After 24h stimulation, reporter activity was analyzed. (D) Wild-type (WT) and pan^-/--AF1AD26^ (pan^-/-^) cells were transfected with Pangolin overexpression vector under the control of the Actin promoter together with *wingful* luciferase reporter expression vector and Renilla expression vector 24 h prior stimulation with WCM (as control CM was used). After 24h stimulation, reporter activity was analyzed.

From the analysis above, we conclude that Arm is absolutely required for both activation and repression of Wnt/Wg target genes and interpret this as evidence against the existence of an Arm-independent Wnt/Wg signaling transcriptional output. Since several alternative interaction partners for beta-catenin have been proposed for the activation and the repression of genes, such as Sox17 [29], Oct4 [28] and Prop1 [17], we next asked whether TCF-independent Wnt/Wg signaling exists. To search for TCF-independent Wnt/Wg signaling, we utilized a similar setup as described above to generate *pan* null mutant cells. Two distinct sgRNAs were used to target independent loci within the *pan* gene (Fig 4A). We isolated a population of *pan* null mutant cells that no longer contain any wild-type allele. Similar to the arm^-/--AFII7/8^ cells, the selected *pan* null mutant cells, termed pan^-/--AF1AD26^, carry three defined mutations that lead to frameshift mutations. Molecular analysis of the alleles revealed no wild-type allele but a large deletion of approximately 9 kb spanning the two selected CRISPR sites (Fig 4B). In addition, pan^-/--AF1AD26^ cells also harbor two distinct frameshift mutations in the HMG box, both of which result in premature termination codons (S3A Fig) and NMD. Consistent with this qRT-PCR analysis showed a reduction of *pan* mRNA in knockout cells compared with wild-type cells (S3B Fig). The presence of the three *pan* mutant alleles suggests that at the *pan* locus Kc cells are polyploid; segmental polyploidy has been reported for Kc cells [40]. Since no anti-Pan antibodies were available to confirm the absence of functional Pan protein we used the *wingful* luciferase reporter assay, an artificial built reporter giving a robust and high Wg-response [41]. Consistent with the absence of Pan, in pan^-/--AF1AD26^ cells the *wingful* reporter could no longer be induced after WCM-stimulation; responsiveness could be restored by Pan overexpression (Fig 4C and 4D).

**Fig 5:**
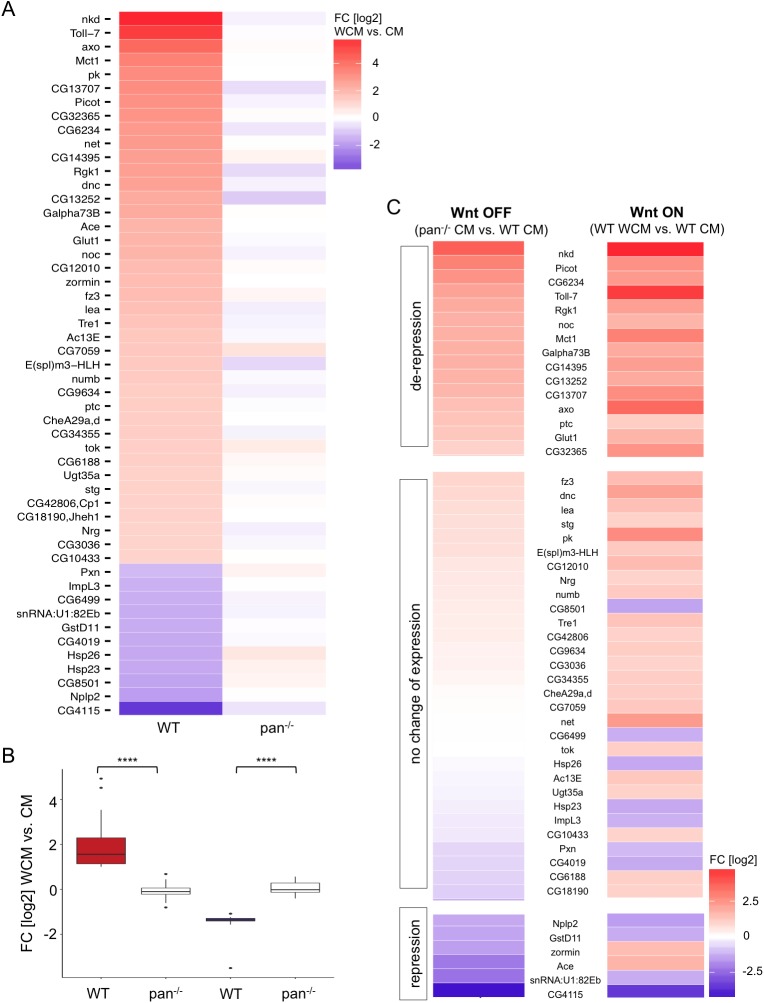
Gene expression analysis of Wnt/Wg target genes in pan^-/--AF1AD26^ cells. (A) Heat map of Wnt/Wg target genes showing their log2 fold change (FC) of expression after WCM treatment versus control treatment in wild-type (WT) and pan^-/--AF1AD26^ (pan^-/-^) cells, respectively. The genes are listed according to their intensity of induction in WT cells. Strongest up-regulated genes are on top. Up-regulated genes are shown in red, down-regulated genes in blue, no expression in white. (B) Boxplots showing the difference in gene activity for up- and down-regulated genes after WCM stimulation in wild-type (WT) and pan^-/--AF1AD26^ (pan^-/-^) cells. Paired t-test: *** ≤ 0.0001. (C) Gene expression analysis of Wnt/Wg target genes in the Wnt OFF and Wnt ON state. Heat map of log2 fold change (FC) according to the genotype (pan^-/--AF1AD26^ (pan^-/-^) CM vs. wild-type (WT) CM) or WCM treatment (wild-type WCM vs. wild-type CM). The genes are listed according to their expression levels in the Wnt OFF state. Up-regulated genes are in red, down-regulated genes in blue, no expression in white. De-repression: fold change (log2) ≥ 1, p-value ≤ 0.0005, repression: fold change (log2) ≤ -1, p-value ≤ 0.0005.

To answer the question of whether Pan is dispensable for Wnt/Wg-regulated induction of target genes, we treated pan^-/--AF1AD26^ cells with either WCM or CM and performed RNA-seq. We observed that pan^-/--AF1AD26^ cells can no longer transduce the Wnt/Wg signal as expression of none of the identified Wnt/Wg targets was altered. Neither positive nor negative Wnt/Wg-target genes significantly changed their expression profile in *pan* knockout cells after Wg stimulation providing evidence that Pan is indispensable for the activation and repression of Wnt/Wg target genes (Fig 5A and 5B). The lack of a change in the expression of several candidate Wnt/Wg targets was confirmed by qRT-PCR (S2 Fig).

### De-repression in the absence of Pan

Like most major developmental signaling pathways, the Wnt/Wg system uses a “transcriptional switch” mechanism to positively regulate target gene expression [42]. In the absence of Wnt/Wg signaling, the transcription of target genes is repressed by Pan via its interaction with co-repressors such as Groucho or Coop [13, 15]. Pan turns into an activator when complexed with Arm following pathway activation. It has been shown that loss of Pan function leads to de-repression of the Wg target genes *nkd* and *CG6234* in the Wg OFF state *in vivo* and *in vitro* [35, 43]. To determine whether this mode of action is valid for the entire set of identified Wnt/Wg target genes we compared the gene expression profiles of wild-type and pan^-/--AF1AD26^ cells in the absence of Wnt/Wg signaling. Interestingly, we found that only a fraction (37.5%) of positive target genes were de-repressed in *pan* null mutant cells (Fig 5C; fold change ≥ 2; p-value ≤ 0.0005); among them were *nkd* and *CG6234* [35]. We also noted that this set of de-repressed genes is highly induced in the presence of Wg ligand (Fig 5C). In contrast, the absence of Pan had no effect on the basal expression of the other (the majority) target genes. However, we also identified some genes exhibiting reduced levels of expression in unstimulated *pan* knockout cells (Fig 5C), suggesting that Pan might be required for their transcription in the absence of Wnt/Wg signaling. Blauwkamp and colleagues (2008) proposed this mode of action for Pan in *Drosophila* Kc cells for several negative target genes, when cells were not exposed to Wnt/Wg [20].

### Genome-wide identification of Wnt/Wg-responsive enhancers

**Fig 6:**
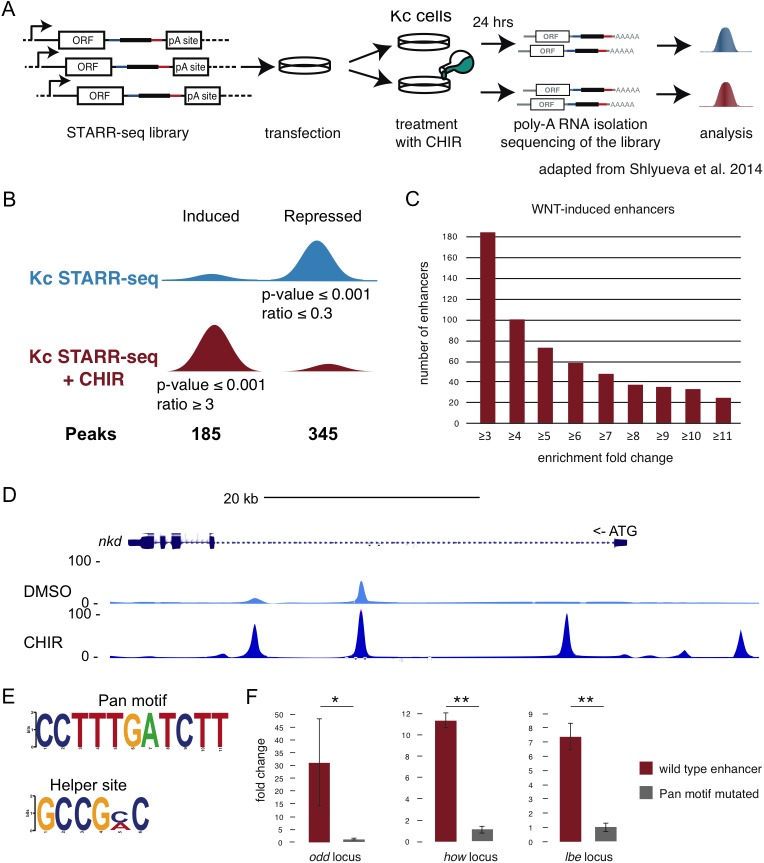
Identification of Wnt/Wg-responsive enhancers. (A) Schematic overview of the STARR-seq experimental setup (adapted from [45]). (B) Cartoon representing STARR-seq peaks that were induced or repressed after activation of the Wnt/Wg pathway and the number of such peaks. (C) Distribution of induced peaks according to their fold induction. (D) UCSC genome browser screenshot of STARR-seq tracks in the *nkd* gene loci. (E) PWM logos for the Pan and Helper motif. (F) Fold induction of normalized luciferase signal for induced enhancers near *odd*, *how* and *lbe* genes. In red: wild-type sequences; in grey: sequences with mutated Pan motif. All DNA sequences are listed in S3 Table. Paired t-test: *p-value = 0.01; **p-value≤10–4. Data are shown as mean ± SD of two experiments.

Transcription factors bind to specific signal responsive elements in the promoters or enhancers of target genes in order to regulate their expression [44]. So far we have analyzed in detail the Wnt/Wg-triggered transcriptional output and demonstrated that both, Arm and Pan are absolutely required for the activation and repression of Wnt/Wg target genes in *Drosophila* cells. However, in order to obtain a more complete understanding of the transcriptional regulation of Wnt/Wg target genes, we carried out Self-transcribing-active-regulatory-region-sequencing (STARR-seq), a genome-wide enhancer activity assay that reveals the identity of DNA sequences that can function as enhancers in a particular cell type [45–46] and in response to external stimuli, such as the insect steroid hormone ecdysone [47]. To identify enhancers whose activity changes in response to the Wnt/Wg signal, we performed STARR-seq under conditions of active Wnt/Wg signaling and under control conditions (Fig 6A, S4A Fig). For technical reasons, we used the Gsk3β-inhibitor CHIR99021 (CHIR)–a widely used alternative inducer of Wnt-signaling to stimulate Wg signaling in the STARR-seq experiments [48, 49] (see Material and Methods), whose activity we compared to WCM by using the *wingful* reporter (S5A Fig). Furthermore, treatment with CHIR robustly induced expression of known Wg targets in *Drosophila* cells (S5B and S5C Fig).

Activation of the Wnt/Wg signaling pathway led to robust changes in enhancer activities: we identified 185 STARR-seq peaks (p-value ≤ 0.001) that were at least 3-fold induced in the CHIR-treated versus control sample, and 348 that were at least 3-fold repressed (Fig 6B). Among the induced peaks, 73 (39.5%) were induced more than 5-fold and 32 (17.2%) more than 10-fold (Fig 6C). We found several enhancers, which have already been described as WREs in *Drosophila* Kc cells. For instance we identified two enhancers close to the TSS of *nkd* (first intron and 10 kb upstream of TSS) (Fig 6D), the well-studied WRE 2.2 kb upstream of the TSS (transcription start site) of *Notum*, an enhancer 15.2 kb upstream of *pxb* and an element in the 5’ intergenic region 178 bp upstream of *Ugt36Bc* [50, 43, 20] (S4B Fig). We validated activated and repressed STARR-seq enhancers in luciferase reporter assays as described in [47]. Consistent with the STARR-seq results, we found luciferase reporter activities responded as expected to both CHIR treatment and WCM treatment: increased activity for activated enhancers and decreased activities for repressed enhancers (S4C and S6 Figs). Taken together, these results indicate that the activities of STARR-seq detected enhancers are modulated by Wnt/Wg signaling.

### The TCF/Pan motif is necessary for Wnt/Wg-induced enhancers

To further test that the identified enhancers were directly regulated by Pan, we assessed the enrichment of known transcription factor motifs [46] in Wnt/Wg-responsive STARR-seq enhancers in comparison to negative control sequences (see Material and Methods). The known TCF/Pan motif [51] (Fig 6E) was strongly enriched in induced enhancers (2.7-fold enrichment, p-value = 1.3x10^-8^), whereas it was not enriched in constitutive or repressed enhancers (p-value = 0.27 and p-value = 0.08, respectively). Using *de novo* motif discovery (see Material and Methods) we found an additional Helper site motif in induced enhancers (GCCGCC, p-value = 3.4x10^-14^; Fig 6E), which is a GC-rich element near TCF/Pan binding sites that is critical for Wnt/Wg target gene activation [52–53, 11]. To experimentally validate the necessity of the TCF/Pan motif for Wnt/Wg induced enhancers, we tested wild-type and mutated versions of the TCF/Pan motif in 3 enhancers of the *odd*, *how* and *lbe* genes in luciferase assays. While the wild-type enhancers activated luciferase reporters 31-, 11- and 7-fold after Wnt/Wg induction by CHIR treatment, the Pan motif-mutant sequences did not respond to treatment (<1.2-fold induction), a substantial and significant difference in each case (p-value≤0.01; Fig 6F), indicating that at least these 3 Wnt/Wg-responsive enhancers require the TCF/Pan motif.

### Pan regulates Wnt/Wg-responsive enhancers

**Fig 7:**
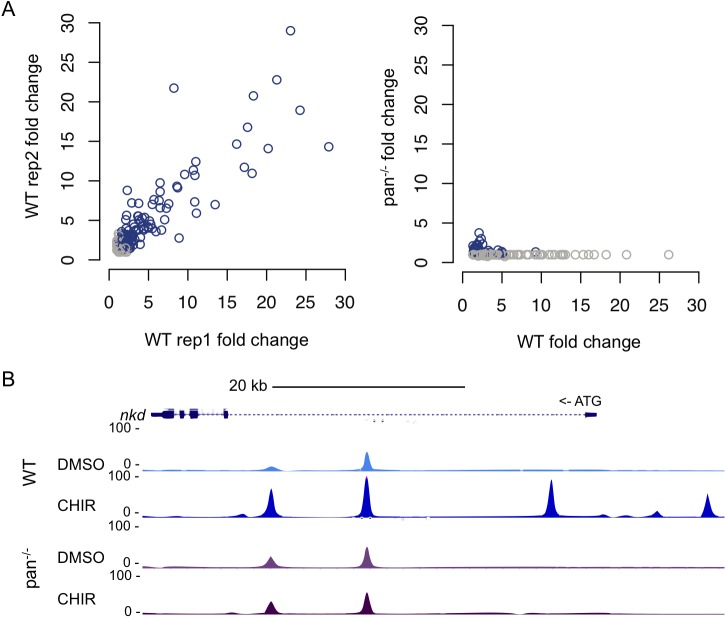
Wnt/Wg-induced enhancers depend on Pan. (A) Scatterplots show signal fold induction at induced enhancers for STARR-seq replicates in wild-type (WT) cells (left) and for comparison of WT versus pan^-/--AF1AD26^ (pan^-/-^) cells (right). Grey dots indicate non-significant induction (p-value>0.001). (B) UCSC browser screenshot of STARR-seq tracks in WT and pan^-/--AF1AD26^ (pan^-/-^) cells for *nkd* gene locus.

Given the enrichment of the TCF/Pan motif in the Wnt/Wg-responsive STARR-seq enhancers and the necessity of this motif for enhancer function, we next examined whether Wnt/Wg-responsive enhancers require the Pan protein. We repeated the STARR-seq experiments in *pan* null mutant cells (S7A Fig) and again confirmed our findings for a subset of the enhancers by treatment with WCM (S6 Fig). Consistent with our analysis of target gene expression by RNA-seq, we found that enhancer-induction was overall strongly reduced from 26.1-fold the highest induction in wild-type cells to at most 3.8-fold in *pan* null mutant cells and that the vast majority (80%) of Wnt/Wg-induced enhancers no longer responded to pathway activation (Fig 7A). For example, the enhancers in first intron and 10 kb upstream of TSS in the *nkd* gene locus that were strongly induced in wild-type cells by Wnt/Wg signaling were not any more induced nor detected in *pan* knockout cells (p-value>0.001, Fig 7B). We confirmed these findings by testing several of the most strongly activated enhancers in luciferase reporter assays. In agreement with the STARR-seq results, enhancers that were strongly activated by Wnt/Wg signaling in wild-type cells did not respond to Wnt/Wg pathway activation in *pan* knockout cells (S7B Fig). Taken together, these results argue that Pan is required for the activation of Wnt/Wg-responsive enhancers.

## Discussion

According to the generally accepted dogma the canonical Wnt signaling pathway culminates in the transcriptional induction of target genes via the beta-catenin/TCF complex. During the past decade, several alternative configurations of the Wnt pathway have been proposed in which either beta-catenin or TCF is bypassed. A recent study explored the co-occupancy of TCF4 and beta-catenin using ChIP-seq and showed that TCF4 is the major factor in tethering beta-catenin to DNA [24]. However, the study could not exclude the possibility that other putative factors could compensate the lack of TCF or beta-catenin–an aspect that is still poorly understood in the field of Wnt research. In the present study, we investigate whether and, if yes, to which extent a Wnt response can bypass beta-catenin or TCF. To this aim we used somatic cell genetics in *Drosophila* cultured cells. As a basis for our analysis, we first carried out a systematic and genome-wide study to explore all Wnt/Wg-related transcriptional outputs in this system. We identified a set of 51 genes that are induced upon Wg stimulation. To probe whether their expression requires Arm or Pan, we generated cells lacking one or the other of these factors using the CRISPR/Cas9 technology. Surprisingly, we found that Arm and Pan are both absolutely required for all Wnt/Wg-related transcriptional outputs in this system. As a transcription factor, Pan binds to DNA regulatory elements up- or downstream of the TSS of its target genes. Thus, next we asked, whether these DNA regulatory elements–enhancers/repressors–are dependent on Pan using STARR-seq. Impressively, consistent with our RNA-seq analysis, we found that the induction of Wnt/Wg-responsive enhancer elements fully depends on Pan.

In our work we identified eleven down-regulated target genes and showed that knockout of Arm or Pan is sufficient to abrogate their repression. We observed the same effect for repressed enhancers in *pan* null mutant cells. These findings are in line with a previous study in *Drosophila* Kc cells [20], in which it was shown that Pan and Arm are required for the repression of the negative target genes *Pxn*, *Ugt36Bc*, *Tig* and *Ugt58Fa* [20]. We also found *Pxn* in our Wnt/Wg target gene set. However, the other genes were less than 2-fold repressed in our system and thus did not pass our selection criteria. This might be due to technical differences in Wnt-pathway stimulation and/or timing. Blauwkamp and colleagues showed also in their study that the negatively regulated targets exhibited lower expression upon Pan reduction in the Wnt OFF state [20], implicating that Pan normally activates their expression even in the absence of Wg ligand. When analyzing our data, we found that only half of the negative target genes appear to be activated in the Wnt OFF state upon Pan abrogation, the remaining targets did not exhibit a significant change in their expression profile. This suggests that they might be indirect targets or independent of Pan. Furthermore, we found that several repressed enhancers possess neither the traditional TCF/Pan binding motif, nor the previously reported alternative binding site important for repression, indicative for a Pan-dependent indirect regulation of repressed enhancers. It is likely that Pan is tethered to the DNA by other co-factors as it was shown for *dpp* or *CDH1* [21, 23]. Thus, these Pan-dependent enhancers without any known TCF/Pan binding site provide a good starting point for further molecular studies to gain insight into the still incomplete model of Wnt-mediated repression [16].

In sum our results demonstrate that all Wnt/Wg-related transcriptional output in *Drosophila* cells requires Arm and Pan and that the induction of Wnt/Wg-responsive enhancers is fully dependent on Pan. Hence, collectively our data argue against the existence of distal branching of the Wnt pathway in this system.

## Materials and methods

### *Drosophila* cell culture

*Drosophila* Kc167 cell lines were cultured in M3+BYPE medium, supplemented with 5% fetal bovine serum (FBS) and 1% penicillin and streptomycin at 25°C.

### Activating Wnt signaling in *Drosophila* Kc cells

Wg-CM was harvested from S2 tubulin wingless cells. S2 tubulin wingless cells were seeded 24 h prior collecting the supernatant (1x10^6^ cells/ml) by centrifuging the cells at 3500 rpm for 5 min. For the control medium S2 cells were prepared as described above. WCM or CM was added to Kc cells for 24 h to induce Wnt/Wg signaling. To induce the Wnt/Wg signaling pathway with CHIR99021 (S1263, Selleckchem), 25 μM of the inhibitor was used and added to the medium for 24h. As control DMSO was used. After 24 h of induction, cells were harvested.

### Cas9 and gRNA plasmids

Cas9 (49330, Addgene) and empty gRNA vector (49410, Addgene) were obtained from Addgene. Oligo design and cloning was accomplished after manufacturer’s protocol.

### Mutagenesis of genes with CRISPR/Cas9

CRISPR was performed as described in [37]. Briefly, cells were plated at 2 x 10^6^ cells per well of a 6-well dish and a total of 1.7 μg DNA, Cas9 and gRNA in a 1:1 ratio, was co-transfected into each well using Fugene HD (Promega) at a 1:2 ratio (μg:μl), following manufacturer’s instructions. Both gene loci were targeted simultaneously using a gRNA and Cas9 with integrated gRNA. Transfections were analyzed after 3 days, and selection was performed in 5 μg/ml Puromycin (P8833 Sigma). The genotype was analyzed using PCR primers spanning the cut site. PCR products were cloned in pGEMT-vector system (Promega) and 10–100 clones were analyzed by sequencing. Primers for gRNA cloning and for detection of CRISPR events are available in the S1 Table.

### Western blot

Nuclear protein extraction was performed as described in [54]. For Western blot analysis, monoclonal anti-Arm (1:500; N2(7A1), DSHB) and monoclonal anti-alpha-Tubulin (1:5000; T5168, Sigma) antibodies were used and followed by HRP-anti-mouse IgG (705-035-003, Jackson Immuno Research Laboraties, inc).

### qRT-PCR

Real-time q-PCR analyses were carried out with SYBR Green Supermix (BioRad) on a iCycler iQ real-time OCR detection system (BioRad). For qRT-PCR, total RNA was extracted from 1–2 x 10^6^ cells with NucleoSpin RNA extraction kit from Macherey-Nagel according to the manufacture’s protocol and reverse transcribed with Roche, followed by qRT-PCR. Sequences of the primer pairs used are listed in S1 Table.

### RNA-seq

All pair-end sequencing was performed on an Illumina HiSeq2500 machine at the Genomics Platform of the University of Geneva. For all experiments we compared three independent biological replicates and merged them for the subsequent analysis. All RNA-seq files are available from SRA NCBI database. Submission code: SUB2472808; Study: PRJNA378604 (Accession Number SRP101692).

### Computational analysis

All deep-sequencing data were mapped to the *Drosophila* reference genome dm3 using TopHat and analyzed as described in [34] and using thresholds as indicated above. We used GraphPad Prism for all statistical analysis and R for plotting.

### STARR-seq

STARR-seq in *Drosophila* WT cells and *pan* knockout cells was performed in two biological replicates as described in [47]. To obtain Wnt-responsive enhancers, cells were treated with 25μM CHIR99021 or DMSO for 24h. Data were analyzed as described in [47]. For Fig 7A fold enrichments were calculated directly over DMSO-treated samples at summits of induced enhancers and p-values indicate significance of the fold change. All STARR-seq files are available at the GEO database (GEO number GSE96542).

### Motif analysis

For TCF/Pan motif enrichment analysis, we used 200 bp regions around the summit of 185 induced, 348 repressed, 1834 constitutive enhancers, and 987 random sequences that were not detected with STARR-seq but followed the same genomic distribution (denoted as negative regions). Enrichments were calculated as described [46]. *De novo* motif analysis was done with DREME using negative regions as a background set (see S2 Table).

### Reporter assay

Enhancer candidates were amplified from genomic DNA of *Drosophila* Kc167 cells (for primers see S3 Table). All candidates were subcloned to either pCR8/GW/TOPO (Invitrogen) or pENTR/TOPO (Invitrogen) and delivered into the firefly luciferase vector [45] using the Gateway LR Clonase II enzyme mix (Invitrogen). Kc cells (1x10^5^) were transfected using Fugene HD (Promega) with a total of 300 ng of various plasmid combinations (1:3 ratio of promoter reporter plasmid to Renilla). Luciferase activities were measured 48 h after transfection and after stimulation with either Wg ligand or CHIR99012 using the Dual-Luciferase Reporter Assay System (Promega). Every experiment was repeated at least twice with three replicates in each independent experiment. Enhancers’ sequences used are listed in S3 Table.

## Supporting information

S1 Fig (A) Schematic representation of potential protein products of Arm in arm^-/--AFII7/8^ (arm^-/-^) cells with premature termination codons (stop), which result from introduced frameshift mutations. (B) Full Western blot analysis from Fig 2. As presented in the blot, no truncated versions of Arm could be detected.
(TIF)

S2 Fig qRT-PCR analysis of (A) positive and (B) negative candidate Wnt/Wg target genes in wild-type (WT), arm^-/--AFII7/8^ (arm^-/-^) and pan^-/--AF1AD26^ (pan^-/-^) cells. Cells were stimulated with WCM or CM for 24 h. Analysis of expression profiles of several Wg target genes after treatment versus control confirmed their induction after WCM stimulation. Fold expression changes of mRNA were calculated by dividing WCM treatment-driven expression values by the expression values obtained with the control treatment.
(TIF)

S3 Fig (A) Schematic representation of potential protein products of Pan in pan^-/--AF1AD26^ (pan^-/-^) cells with premature termination codons (stop) due to introduced frameshift mutations. (B) qRT-PCR analysis of *pan* mRNA level with primer targeting its N-term (see S1 Table) in wild-type (WT) and pan^-/--AF1AD26^ (pan^-/-^) cells. Cells were stimulated with WCM or CM for 24 h. Fold expression changes of mRNA were calculated by dividing WCM treatment-driven expression values by the expression values obtained with the control treatment.
(TIF)

S4 Fig (A) Scatterplots of replicates of STARR-seq in wild-type (WT) cells treated with DMSO or CHIR99021 (CHIR). (B) UCSC browser screenshot of STARR-seq tracks in WT cells for *pxb*. (C) Validation of peaks from the constitutive, induced, and repressed enhancer classes by luciferase assays. Log2 fold induction (CHIR-treated versus control) of normalized luciferase signal is shown. Wilcoxon rank-sum test: **p-value = 0.0007, *p-value = 0.003, n indicates the number of enhancers in each group.
(TIF)

S5 Fig CHIR99021 activates reliably and efficiently Wnt/Wg target genes in Drosophila cells. (A) Titration of CHIR99021 in *Drosophila* S2R+ cells. S2R+ cells were transfected with *wingful* luciferase reporter vector and Renilla. Red bars: promoter activation with 25 μM CHIR is as efficient as with Wg ligand. In green is the *wingful* promoter activity after stimulation with ArmS10 depicted, black bar shows the activity after control treatment, grey bars represent the activity after respective CHIR99021 concentration. (B, C) qRT-PCR analysis of gene expression in *Drosophila* Kc cells in the Wnt OFF and ON state. Fold change of gene expression levels were calculated using expression values after WCM (A) or CHIR (B) treatment versus control treatments. Stimulation with WCM and CHIR leads to a similar robust expression of target genes *nkd*, *fz3* and *Toll-7* in wild-type cells.
(TIF)

S6 Fig Validation of (A) induced and (B) repressed candidate STARR-seq enhancers with WCM. Candidate enhancer sequences were cloned into the STARR-seq library luciferase vector, see Material and Methods. Wild-type (WT) and pan^-/--AF1AD26^ (pan^-/-^) cells were transfected with the candidate luciferase reporter expression vector and Renilla expression vector 24 h prior stimulation with WCM (as control CM was used). After 24h stimulation, reporter activity was analyzed.
(TIF)

S7 Fig (A) Scatterplots of replicates of STARR-seq in pan^-/--AF1AD26^ (pan^-/-^) cells treated with DMSO or CHIR99021 (CHIR). (B) Validation of candidate STARR-seq enhancers. Candidate enhancer sequences were cloned into the STARR-seq library luciferase vector, see Material and Methods. Wild-type (WT) and pan^-/--AF1AD26^ (pan^-/-^) cells were transfected with the candidate luciferase reporter expression vector and Renilla expression vector 24 h prior stimulation with CHIR (as control DMSO was used). After 24h stimulation, reporter activity was analyzed.
(TIF)

S1 Table Primer sequences for qRT-PCR, cloning of gRNAs, PCR. (XLSX)

S2 Table *de novo* motif search using DREME. (XLSX)

S3 Table Primer sequences for STARR-seq enhancer validations and Pan motif validation. (XLSX)
