# Supplementary figures and images for: Probing the canonicity of the Wnt/Wingless signaling pathway
Source: PLoS Genet. 2017 Apr 3;13(4):e1006700. doi: 10.1371/journal.pgen.1006700 (PMC5393890; doi:10.1371/journal.pgen.1006700)

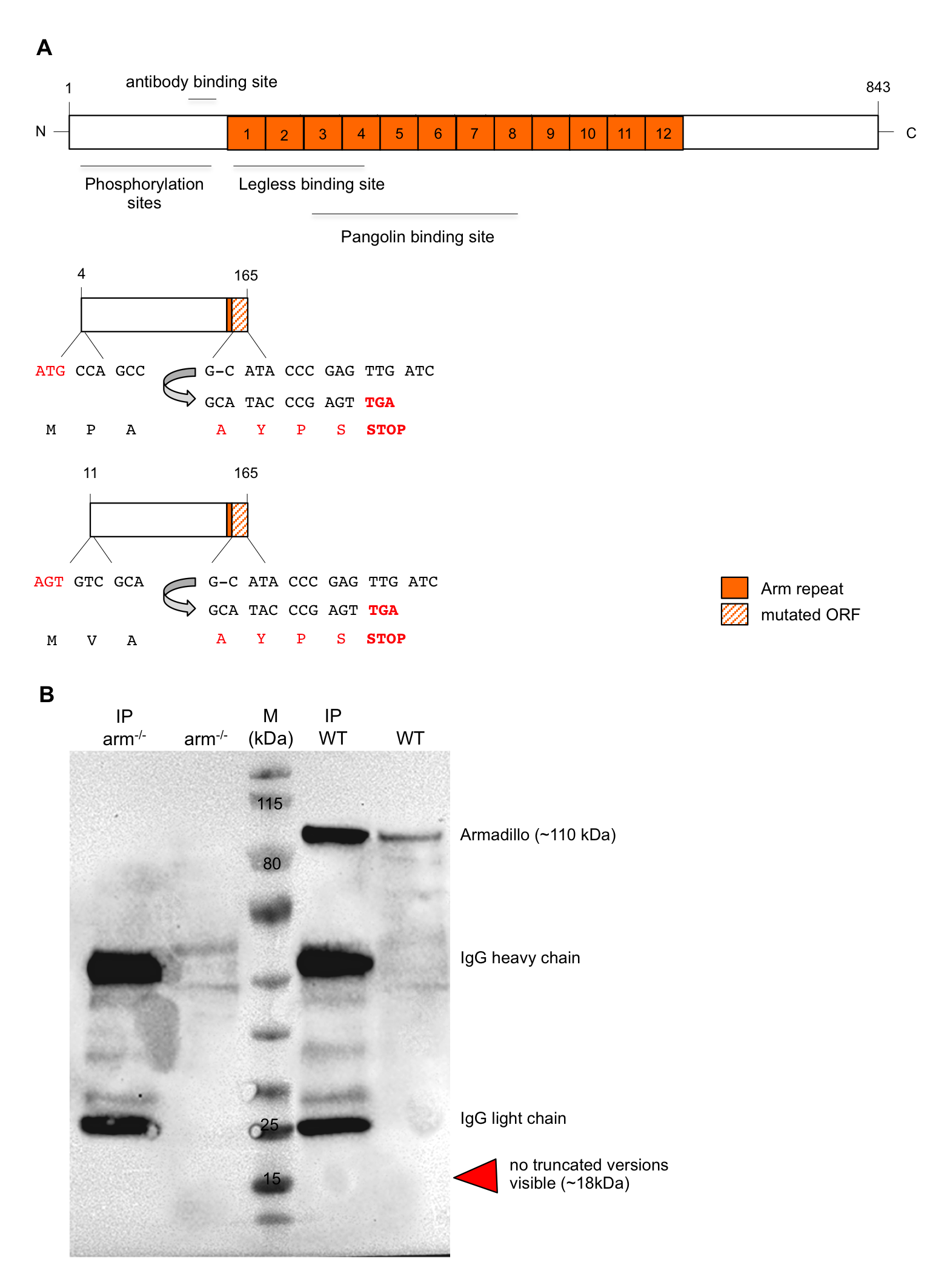

Supplement: S1 Fig — (A) Schematic representation of potential protein products of Arm in arm-/--AFII7/8 (arm-/-) cells with premature termination codons (stop), which result from introduced frameshift mutations. (B) Full Western blot analysis from Fig 2. As presented in the blot, no truncated versions of Arm could be detected. (TIF) [file pgen.1006700.s001.tif]

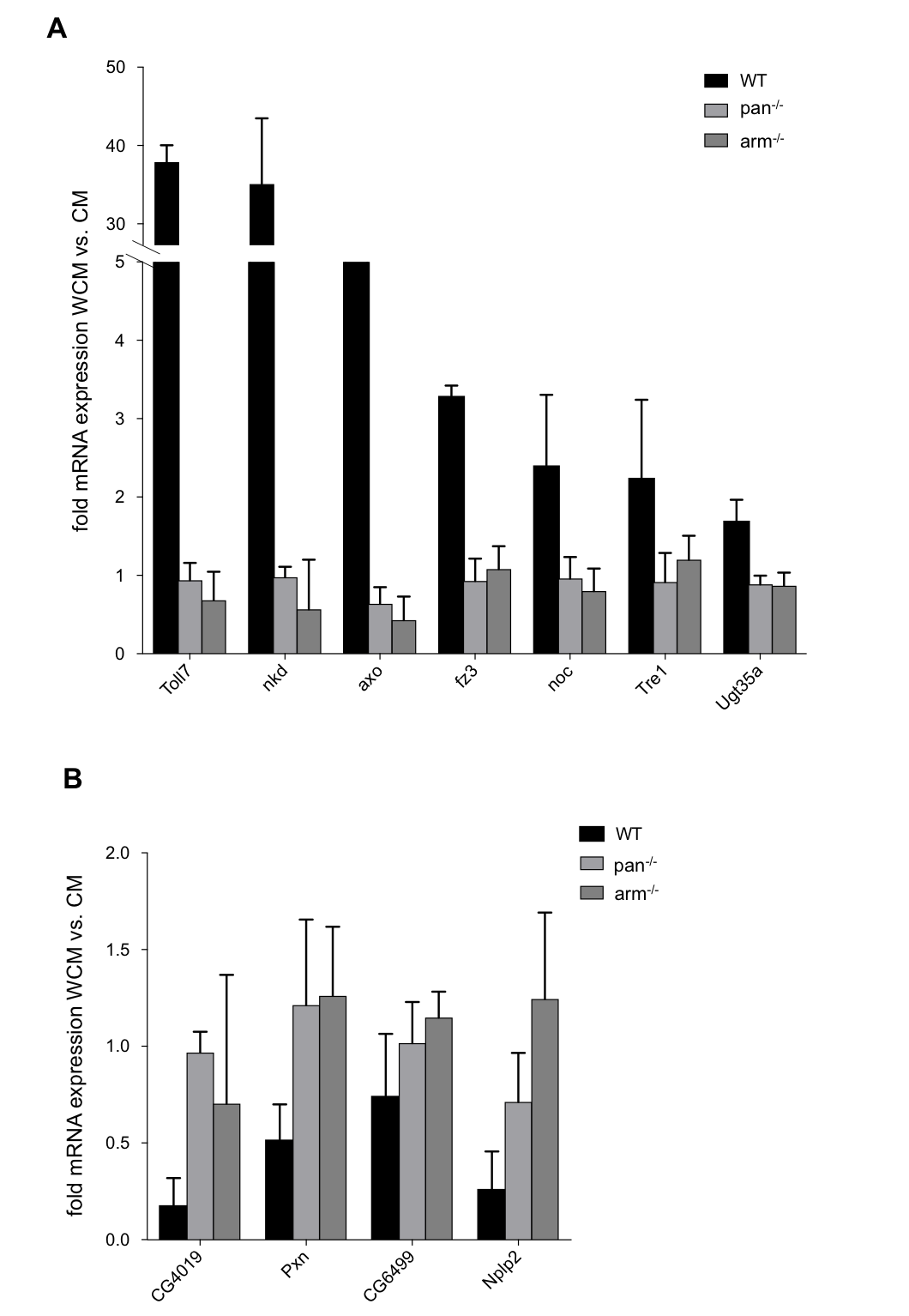

Supplement: S2 Fig — qRT-PCR analysis of (A) positive and (B) negative candidate Wnt/Wg target genes in wild-type (WT), arm-/--AFII7/8 (arm-/-) and pan-/--AF1AD26 (pan-/-) cells. Cells were stimulated with WCM or CM for 24 h. Analysis of expression profiles of several Wg target genes after treatment versus control confirmed their induction after WCM stimulation. Fold expression changes of mRNA were calculated by dividing WCM treatment-driven expression values by the expression values obtained with the control treatment. (TIF) [file pgen.1006700.s002.tif]

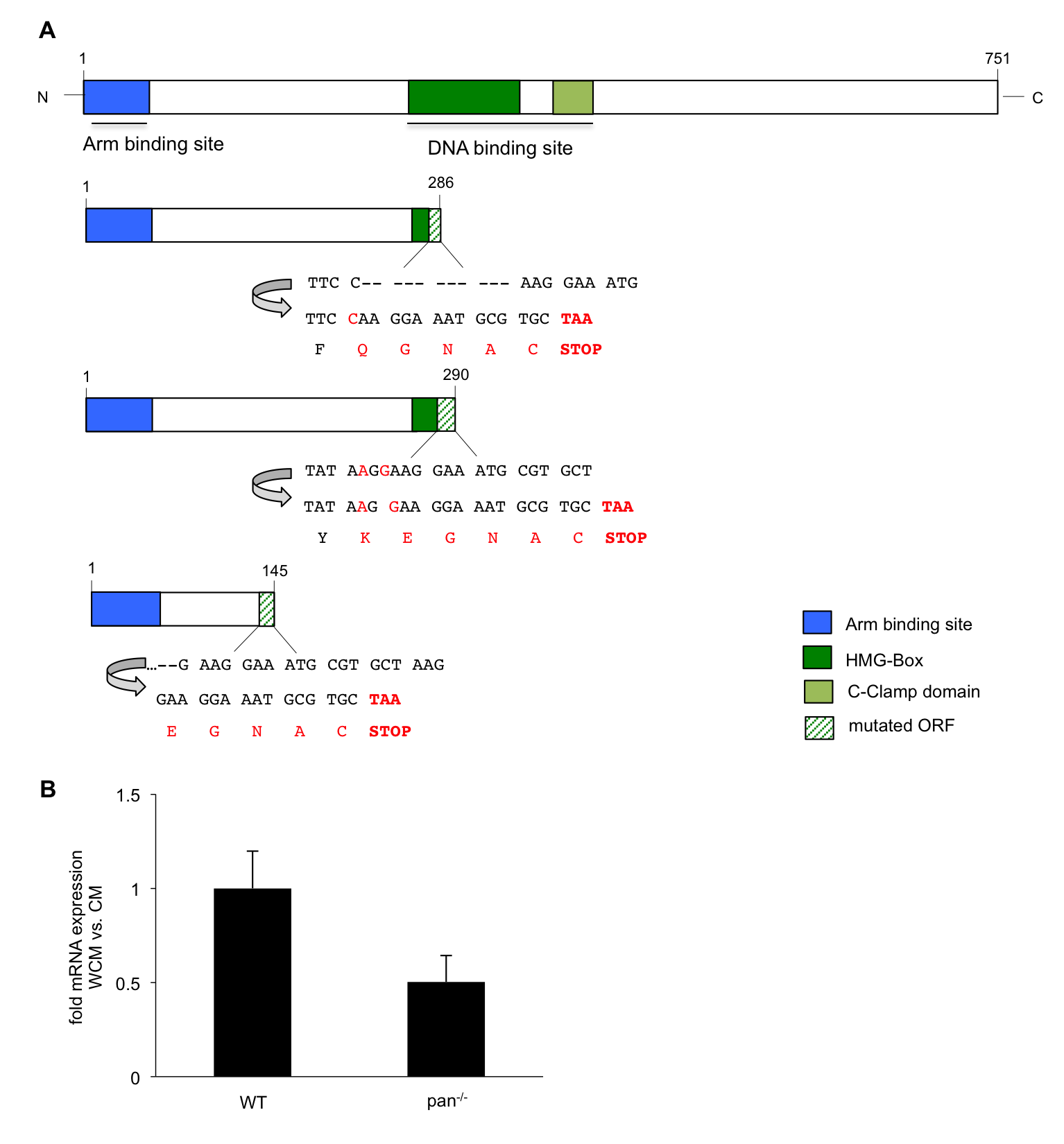

Supplement: S3 Fig — (A) Schematic representation of potential protein products of Pan in pan-/--AF1AD26 (pan-/-) cells with premature termination codons (stop) due to introduced frameshift mutations. (B) qRT-PCR analysis of pan mRNA level with primer targeting its N-term (see S1 Table) in wild-type (WT) and pan-/--AF1AD26 (pan-/-) cells. Cells were stimulated with WCM or CM for 24 h. Fold expression changes of mRNA were calculated by dividing WCM treatment-driven expression values by the expression values obtained with the control treatment. (TIF) [file pgen.1006700.s003.tif]

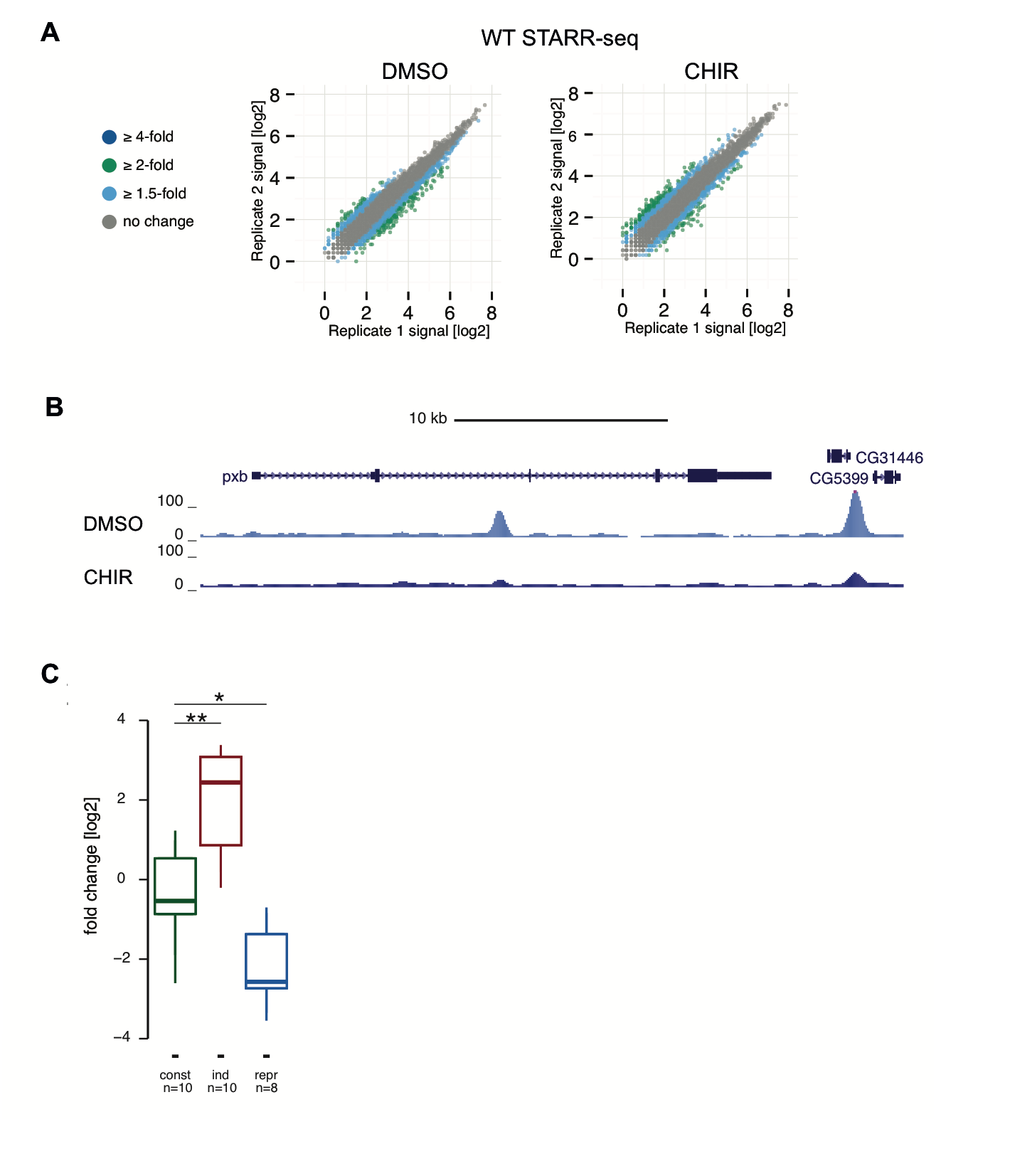

Supplement: S4 Fig — (A) Scatterplots of replicates of STARR-seq in wild-type (WT) cells treated with DMSO or CHIR99021 (CHIR). (B) UCSC browser screenshot of STARR-seq tracks in WT cells for pxb. (C) Validation of peaks from the constitutive, induced, and repressed enhancer classes by luciferase assays. Log2 fold induction (CHIR-treated versus control) of normalized luciferase signal is shown. Wilcoxon rank-sum test: **p-value = 0.0007, *p-value = 0.003, n indicates the number of enhancers in each group. (TIF) [file pgen.1006700.s004.tif]

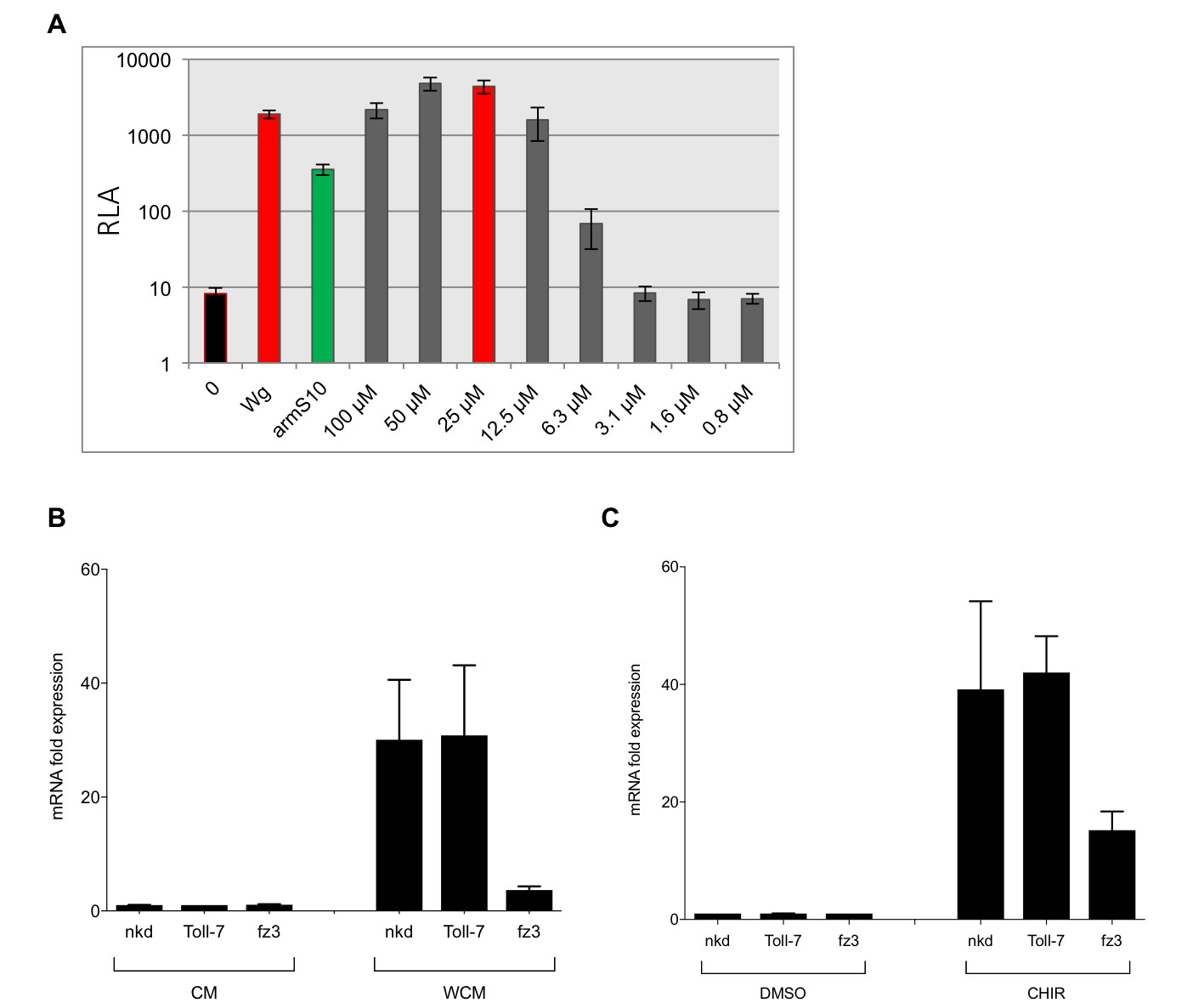

Supplement: S5 Fig — (A) Titration of CHIR99021 in Drosophila S2R+ cells. S2R+ cells were transfected with wingful luciferase reporter vector and Renilla. Red bars: promoter activation with 25 μM CHIR is as efficient as with Wg ligand. In green is the wingful promoter activity after stimulation with ArmS10 depicted, black bar shows the activity after control treatment, grey bars represent the activity after respective CHIR99021 concentration. (B, C) qRT-PCR analysis of gene expression in Drosophila Kc cells in the Wnt OFF and ON state. Fold change of gene expression levels were calculated using expression values after WCM (A) or CHIR (B) treatment versus control treatments. Stimulation with WCM and CHIR leads to a similar robust expression of target genes nkd, fz3 and Toll-7 in wild-type cells. (TIF) [file pgen.1006700.s005.tif]

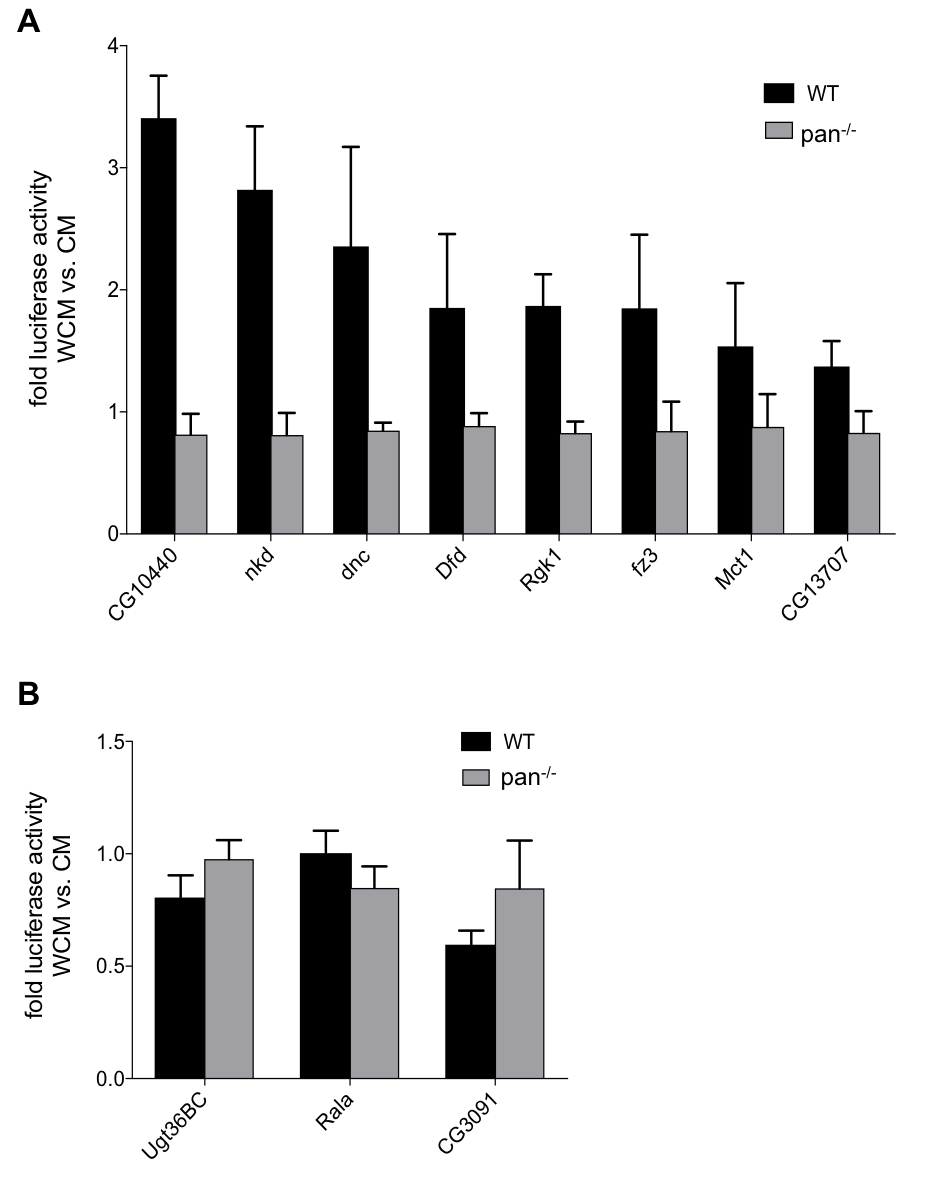

Supplement: S6 Fig — Validation of (A) induced and (B) repressed candidate STARR-seq enhancers with WCM. Candidate enhancer sequences were cloned into the STARR-seq library luciferase vector, see Material and Methods. Wild-type (WT) and pan-/--AF1AD26 (pan-/-) cells were transfected with the candidate luciferase reporter expression vector and Renilla expression vector 24 h prior stimulation with WCM (as control CM was used). After 24h stimulation, reporter activity was analyzed. (TIF) [file pgen.1006700.s006.tif]

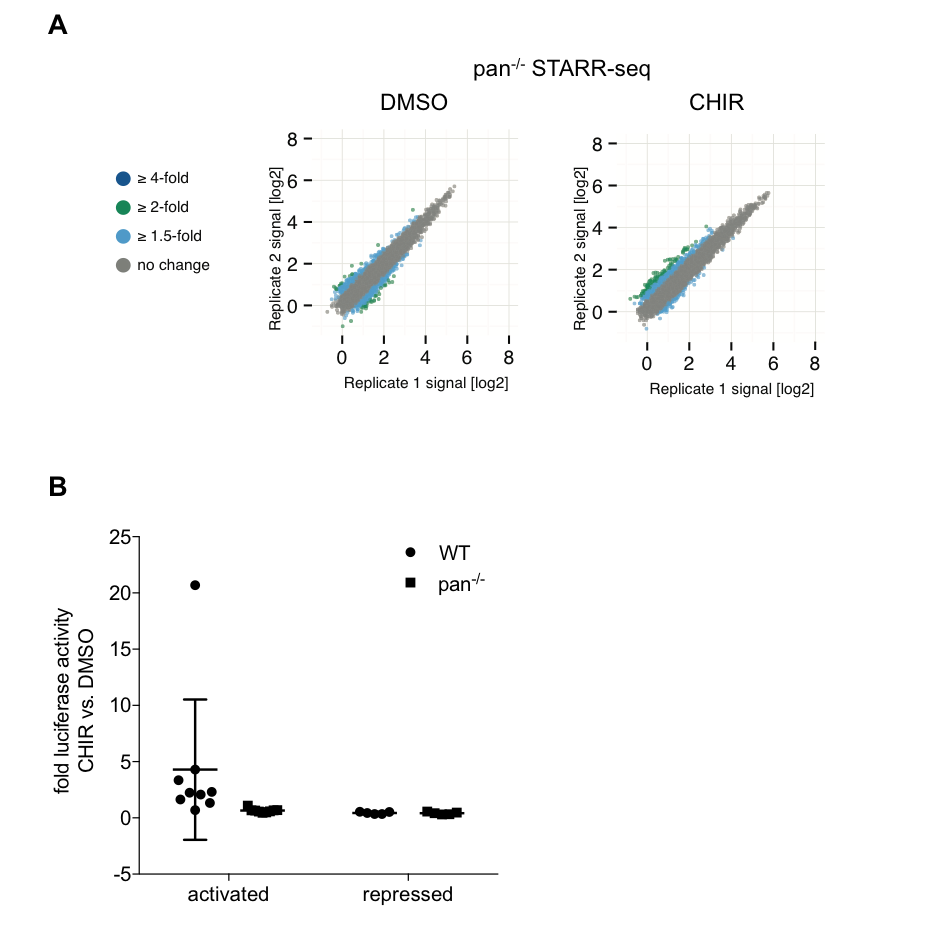

Supplement: S7 Fig — (A) Scatterplots of replicates of STARR-seq in pan-/--AF1AD26 (pan-/-) cells treated with DMSO or CHIR99021 (CHIR). (B) Validation of candidate STARR-seq enhancers. Candidate enhancer sequences were cloned into the STARR-seq library luciferase vector, see Material and Methods. Wild-type (WT) and pan-/--AF1AD26 (pan-/-) cells were transfected with the candidate luciferase reporter expression vector and Renilla expression vector 24 h prior stimulation with CHIR (as control DMSO was used). After 24h stimulation, reporter activity was analyzed. (TIF) [file pgen.1006700.s007.tif]
